# Development of an indirect ELISA for detecting *Toxoplasma gondii* IgG antibodies based on a recombinant TgIMP1 protein

**DOI:** 10.1371/journal.pntd.0012421

**Published:** 2024-08-14

**Authors:** Hongjie Dong, Junmei Zhang, Qi Wang, Yanmei Shen, Beibei Zhou, Lisha Dai, Wenju Zhu, Hang Sun, Xiaoman Xie, Huanhuan Xie, Chao Xu, Guihua Zhao, Kun Yin

**Affiliations:** 1 Shandong Institute of Parasitic Diseases, Shandong First Medical University & Shandong Academy of Medical Sciences, Jining, People’s Republic of China; 2 Digestive Disease Hospital of Shandong First Medical University, Shandong First Medical University & Shandong Academy of Medical Sciences, Jining, People’s Republic of China; University of Texas at El Paso, UNITED STATES OF AMERICA

## Abstract

*Toxoplasma gondii* (*T*. *gondii*) is widely spread around the world, which can cause serious harm to immunosuppressed patients. Currently, the commercial test kits are poor at assessing *T*. *gondii* infection and vaccine effectiveness, making an urgent need to exploit effective enzyme-linked immunosorbent assay with great performance to compensate for this deficiency. Here, the TgIMP1 recombinant protein was expressed in *E*. *coli* BL(21) cells. The TgIMP1 was purified with affinity chromatography and the reactivity was retained with anti-TgIMP1 antibodies. The TgIMP1 was then used to develop an indirect ELISA (IMP1-iELISA) and the reaction conditions of IMP1-iELISA were optimized. As a result, the cut-off value was determined to be 0.2833 by analyzing the OD_450nm_ values of forty *T*. *gondii*-negative sera. The coefficient of variation of 6 *T*. *gondii*-positive sera within and between runs were both less than 10%. The IMP1-iELISA was non-cross-reactive with the sera of cytomegalovirus, herpes virus, rubella virus, *Cryptosporidium* spp., *Theileria* spp., *Neospora* spp. and *Plasmodium* spp.. Furthermore, the sensitivity and specificity of IMP1-iELISA were 98.9% and 96.7%, respectively, based on testing 150 serum samples. The results suggest that this IMP1-iELISA is specific, sensitive, repeatable and can be applied to the detection of T. gondii infections in the medical and health industries.

## Author summary

*Toxoplasma gondii* is an extremely widespread and a worldwide health problem protozoon, which can induce acute and chronic infection and cause severe toxoplasmosis. However, there is still no effective vaccine to prevent infection, drugs used to treat the acute infected individuals have a lot of limitations, and no drugs to treat chronic infected individuals. Indirect ELISA, as a commonly used clinical test with satisfactory detection performance and easy implementation for high throughput testing, has been used to detect various pathogenic microorganisms. Immune mapped protein1 (IMP1) has been identified as a protein that induces a strong immune response in the host. In this study, we obtained IMP1 protein with high purity and used it as coated antigen to prepare an indirect ELISA reagent, and then verified its detection performance (sensitivity, specificity, repeatability). This study enriches the diagnosis of toxoplasmosis and provides strong technical support for epidemiological investigation and transmission control of toxoplasmosis.

## 1. Introduction

*Toxoplasma gondii* (*T*. *gondii*) is the most successful parasite in the world, and toxoplasmosis, the zoonotic disease caused by the parasite infects 30–50% of the global population [1, 2]. As one of the world’s three major foodborne pathogens, *T*. *gondii*’s intermediate hosts include humans, rodents, domestic animals, and other carnivorous animals, with the definitive host being cats [3]. After infection, *T*. *gondii* can cross the placental barrier and blood-brain barrier of the human body, causing serious clinical diseases in immunocompromised people (such as organ transplant patients, AIDS patients, the elderly, children, pregnant women, etc.), and can also cause serious eye diseases or mental and behavioral disorders in normal adults [4, 5]. In view of the serious potential hazards and economic losses, it is particularly critical to develop a diagnostic method for toxoplasmosis with excellent performance for monitoring the epidemic situation.

Toxoplasmosis diagnostic methods include pathogenic tests, imaging tests, immunological tests, and molecular testing [6]. Pathogenic tests involve repeated transfection and killing of large number of test animals [7]. The procedure is complicated and unsuitable for large-scale detection in humans and laboratory animals [8]. Imaging tests, which have relatively low accuracy and are prone to misdiagnosis, must be combined with additional detection methods [9]. Molecular testing is the most sensitive, but requires specific amplification instruments and are susceptible to aerosol contamination [10]. Immunological tests (the detection of serum specific antibody IgG/IgM) has become a widely used detection method due to its elevated sensitivity and strong specificity, and among which indirect ELISA (iELISA) is the most predominant method [11]. The diagnostic antigens used in iELISA include soluble extract of *T*. *gondii* tachyzoites (surface proteins SAG1, SAG2, etc.), excreted and secreted antigens (MIC3, GRA1, GRA7, etc.) and various recombinant antigens [12–14]. However, the soluble extracts of *T*. *gondii* tachyzoites have a complex composition and are less specific. The solubility of excreted and secreted antigens is poor. In addition, the expression of common antigens in the physiological cycle of *T*. *gondii* is short-lived and not suitable for the detection of long-term chronic infection. Therefore, the performance of antibody detection kits developed based on the *T*. *gondii* protein mentioned above needs to be further validated. Rats and mice, commonly used in laboratories, are common intermediate hosts of *T*. *gondii*. Due to their general susceptibility, they have been used to construct models of acute/chronic animal infections of *T*. *gondii* and to develop novel control measures [15]. Currently, kits that can be used to detect toxoplasmosis in laboratory animals are rare, expensive and on the verge of being discontinued, impeding scientific research into toxoplasmosis. There is an urgent need to develop a new low-cost and high-sensitivity method for the *T*. *gondii* detection.

Immune mapped protein1 (IMP1) is an ideal candidate antigen first identified by Blake et al. in 2011 [16]. It has been proven to be strongly immunogenic to chicken coccidiosis [17]. It is persistently and robustly expressed in *E*. *tenella*, *T*. *gondii*, and *Neospora caninum*, and is a protective antigen shared by different coccidial parasites [18, 19]. Meanwhile, Blake et al. also confirmed that IMP1 can be expressed in vivo and still have excellent immunogenicity in attenuated strains of *T*. *gondii* ME49 [16]. The potential of IMP1 protein expression products as vaccine candidate antigens has been demonstrated. The pcDNA3.1-IMP1 nucleic acid vaccine constructed by Cui X et al. showed significant immunological protection against immunocompromised mice [20]. The ETIMP1-VFLIC subunit vaccine constructed by Yin et al. also showed that IMP1 is synergistic and can cooperate with other antigens or act as an adjuvant to exert immunological efficacy [21]. Due to the strong immunogenicity and persistent expression of IMP1, a *T*. *gondii* IgG indirect ELISA based on soluble TgIMP1 (IMP1-iELISA) was developed, which may provide a tool for advancing understanding of toxoplasmosis epidemiology.

## 2. Materials and methods

### 2.1 Ethics statement

All of animal experimentation were carried out strictly according to the International Guiding Principles for Biomedical Research Involving Animals, as issued by the Council for the International Organizations of Medical Sciences and the guidelines set by the Institutional Animal Care and Use Committee of Shandong First Medical University (approval number: w202103030088). The study was carried out in compliance with the Animal Research: Reporting of In Vivo Experiments (ARRIVE) guidelines.

### 2.2 *T*. *gondii* strain, test animal, plasmid, expression strains

Kunming mice of 5–6 weeks were purchased from Pengyue Laboratory Animal Management Co., Ltd (Jinan, China). The passage and preservation of *T*. *gondii* tachyzoites (RH, WH6, ME49 and PRU strains) were completed in our laboratory. *E*. *coli* BL(21) competent cells and expression strains were purchased from Takara Biomedical Technology Co., Ltd (Dalian, China). The prokaryotic expression plasmid pET-28b was stored in our laboratory. The *T*. *gondii*-positive and *T*. *gondii*-negative serum were obtained from infected mice and uninfected mice, respectively.

### 2.3 Experiment reagents

The M5 HiPer Universal Plus RNA Mini Kit (ref # JK-R5292) was purchased from Mei5 Biotechnology Co., Ltd. (Beijing, China). The RevertAid First Strand cDNA Synthesis Kit was purchased from Thermo Fisher Scientific (Waltham, MA, USA). The plasmid extraction kit was purchased from Sangon Biotech Co., Ltd. (Shanghai, China). Diaminobenzidine Horseradish Peroxidase Color Development kit, HRP-labeled SPA, Bovine serum albumin (BSA) and standard calf serum were purchased from Boster Biological Technology Co., Ltd. (Wuhan, China). The 1×ELISA coating buffer was purchased from Coolaber Technology Co., Ltd. (Beijing, China). The rabbit anti-TgIMP1 antibodies used for the western blot were obtained by immunizing rabbits with purified TgIMP1 and then purified from serum. The HRP-conjugated Affinipure Goat Anti-Mouse IgG (ref # SA00001-1) and HRP-conjugated Affinipure Goat Anti-Rabbit IgG (ref # SA00001-2) were purchased from Proteintech Group, Inc (Wuhan, China). The *T*. *gondii* indirect hemagglutination test (IHA) kit was purchased from Shouyan Biotechnology Co., Ltd. (Lanzhou, China). The commercial IgG ELISA kit for detecting *T*. *gondii* infection was purchased from Xishan Biotechnology Co., Ltd. (Suzhou, China). The mouse IgG ELISA kits for detecting the infection of cytomegalovirus (CMV), herpes virus (HSV), rubella virus (RV), *Cryptosporidium* spp., *Theileria* spp. and *Plasmodium* spp. were purchased from Sipersen Biotechnology Co., Ltd. (Shanghai, China). The mouse IgG ELISA kit for detecting *Neospora* spp. infection was purchased from Fantai Biotechnology Co., Ltd. (Shanghai, China).

### 2.4 RNA extraction and cDNA synthesis of *T*. *gondii*

The tachyzoites of *T*. *gondii* RH strain were retrieved from -80°C storage and rapidly thawed at 37°C, then diluted with normal saline. The concentration of tachyzoites was adjusted to 10^5^/mL, and the female Kunming mice of 5–6 weeks were infected at a dose of 0.3 mL/mouse intraperitoneally. 3–5 days after infection, when the mice showed the typical symptoms of *T*. *gondii* infection, such as reduced food intake, decreased activity, eye closure, hair erection, and tremor, they were anesthetized with ether and immediately dissected in a sterile workbench. The body surface of the mice was disinfected with 75% alcohol, and then the abdominal skin was cut open and 3ml sterile saline was injected. Before pulling out the needle, gently massage the abdomen and then suck the fluid out of the abdominal cavity. Be careful not to puncture the bowel or liver with the needle to avoid bleeding or contamination. After taking a small amount of ascites and examining the *T*. *gondii* with a microscope, the parasite was washed by centrifugation (1200 g, 5 min) and suspension. The parasite was suspended with 1mL of sterile saline, and the infection was re-injected intraperitoneally at the previous dose. This cycle was repeated three times for *T*. *gondii* transmission through mice. The final collected ascites was used for total NRA extraction with the M5 HiPer Universal Plus RNA Mini Kit. *T*. *gondii* RH strain tachyzoite cDNA was prepared using the RevertAid First Strand cDNA Synthesis Kit.

### 2.5 Construction of prokaryotic recombinant expression plasmid

According to the *IMP1* gene sequence (JN657189.1) of *T*. *gondii* virulent RH strain tachyzoite, after analysis by Lasergene software, primers with a length between 18–30 bp, four evenly distributed bases, G and C base content between 40%-60%, Tm value between 55–60°C, and no local dimer or hairpin structure were selected for amplifying the *IMP1* gene. The two specific PCR primers (**S1 Table**) were synthesized by synthesized by Jinan Biosune Biotechnology Co., Ltd (Jinan, China).

The PCR reaction system consisted of 5 μL 10 × Pyrobest Buffer II, 1 μL Pyrobest DNA Polymerase, 4 μL dNTP Mixture (each 2.5 mM), 2 μL forward primer (10 μM), 2 μL reverse primer (10 μM), 1 μL tachyzoite cDNA of *T*. *gondii* RH strain (0.2 ug), 1.6 μL dimethyl sulfoxide (DMSO) and 33.4 μL nuclease-free water. The PCR procedure settings were as follows: pre-denaturation at 95°C for 5 min, denaturation at 94°C for 30 s, annealing at 56°C for 30 s and elongation at 72°C for 90 s, 30 cycles.

The PCR amplification efficiency were identified by 1% agarose gel electrophoresis. The PCR products and pET-28b vector were recovered after treatment with Nde I and Xho I restriction enzymes and then ligated according to a 1: 8 molar ratio between the vector and the target fragment. 5–10 μL ligation product was added into the *E*. *coli* BL21 (DE3) competent cells for DNA transformation. Positive clones were identified by DNA sequencing and double enzyme digestion. Plasmid DNA was extracted by using Plasmid Mini Kit and saved at -20°C for future use.

### 2.6 Protein expression and purification of TgIMP1

For the protein expression of TgIMP1 recombinant protein, *E*. *coli* BL21(DE3) cells containing expression plasmids were grown in LB medium supplemented with 50 μg/mL kanamycin at 37°C, with shaking at 2.5 g. When the OD_600nm_ reached 0.8, the temperature was lowered to 20°C and then a final concentration of 0.3 mM of Isopropyl β-D-Thiogalactopyranoside (IPTG) was added for overnight induction.

For the protein purification, cells were harvested by centrifugation at 5000 g for 20 min. Cell pellet was resuspended in lysis buffer (25 mM Tris-HCl pH 8.0, 150 mM NaCl), and then lysed by sonication on ice. After centrifugation at 28370 g for 50 minutes at 4°C, the supernatant was loaded onto a nickel chelating sepharose affinity column equilibrated with lysis buffer in advance. The column was then washed with wash buffer (25 mM Tris-HCl pH 8.0, 500 mM NaCl, 15 mM imidazole) and then eluted with elution buffer (25 mM Tris-HCl pH 8.0, 100 mM NaCl, 250 mM imidazole). Additional purification was carried out by size-exclusion chromatography using Superdex 200 in 25 mM Tris-HCl pH 8.0, and 500 mM NaCl. Finally, the protein purity was evaluated by SDS-PAGE.

### 2.7 Western blot analysis

The TgIMP1 recombinant protein were resuspended with 50 μL 1 × SDS-PAGE loading buffer and boiled for 10 min before fractionated by electrophoresis on 12.5% SDS-PAGE gels, and the resolved proteins were transferred onto polyvinylidene fluoride membranes. After blocking with 5% skim milk, the membranes were probed with rabbit anti-TgIMP1 antibody (1:700 dilution in TBST) at 4°C overnight, and horseradish peroxidase (HRP)-conjugated goat anti-rabbit polyclonal antibody (1:6000 dilution in TBST) was used as the secondary antibody. The blots were visualized using the ECL reagent (Proteintech; ref # PK10002) according to the manufacturer’s instructions.

### 2.8 Preparation of mice serum

The *T*. *gondii* RH strain causes acute infection in mice, resulting in death within a short period of time during which no specific IgG antibodies are produced. Therefore, we infected mice with ME49, PRU and WH6 strains, which are less virulent, to produce chronic infection.

A total of 90 (45 males and 45 females) Kunming mice aged 5–6 weeks were infected with *T*. *gondii* by intraperitoneal injection of 3×10^4^ tachyzoites (30 mice each for ME49, PRU and WH6 strains). After six weeks of infection, the mice showed obvious symptoms such as stooping, bristling, trembling and reduced activity. The blood was then taken from the posterior ocular venous plexus and centrifuged at 1200 g for 10 min to isolate the supernatant and obtain positive serum. 60 mice were injected with the same volume of PBS and the negative serum was obtained under consistent separation conditions. So we ended up with 90 *T*. *gondii*-positive serum and 60 *T*. *gondii*-negative serum. The serums were stored at -80°C after packaging.

### 2.9 TgIMP1 protein based-indirect ELISA development

Purified TgIMP1 was diluted into five concentration gradients (40 μg/mL, 20 μg/mL, 10 μg/mL, 5 μg/mL and 2.5 μg/mL) with antigen coating buffer for 96-well plate, 100 μL/well. After coating overnight at 4°C, the coating solution was discarded, and the well was washed with PBST (0.05%Tween20 + 0.01 mol/L PBS). Added 200 μL blocking solution containing 10% calf serum to each well, blocked at 37°C for 2 h, and washed with PBST 3 times. *T*. *gondii*-positive sera and negative sera were diluted with PBST at the ratios of 1:25, 1:50, 1:100, 1:200, 1:400, and 1:800, respectively, and then added to ELISA plate with 100 μL/ well. After incubation at 37°C for 1 h, the HRP-conjugated affinipure goat anti-mouse IgG was added to wells (100 μL/well). After incubation at 37°C for 45 min, adding substrate chromogenic solution (TMB) to wells, the OD_450nm_ value of each well was determined after 20 min at room temperature in the dark. The optimal concentration of the coated antigen and the optimal dilution of the serum sample were determined using the checkerboard titration method as described previously with some modification [22]. The optimal coating conditions were screened from the following four schemes. Scheme 1: overnight at 4°C; scheme 2: 37°C for 2 h; scheme 3: 37°C for 30 min, then overnight at 4°C; scheme 4: 37°C for 1 h, then overnight at 4°C.

The blocking solution was selected from 10% calf serum, 5% skim milk and 1% BSA. The four blocking schemes were set as follows: scheme 1: 4°C overnight; scheme 2: 37°C for 2 h; scheme 3: 37°C for 1 h; scheme 4: 37°C for 1 h, then 4°C for 4 h. The optimal blocking solution and blocking scheme were determined through the checkerboard titration method. Five working concentration gradients of the HRP-conjugated affinipure goat anti-mouse IgG (1:2000, 1:3000, 1:5000, 1:8000 and 1:10000) were set for IMP1-iELISA detection.

### 2.10 Determination of the cut-off value for IMP1-iELISA

Forty *T*. *gondii*-negative serum samples were tested using the IMP1-iELISA as described above to determine the cut-off value. Each sample was tested three times. The mean ($\bar{x}$) and standard deviation (SD) of OD_450nm_ values of *T*. *gondii*-negative serum samples were calculated. When OD_450nm_ value ≥ $\bar{x}$+3SD, positive serum samples were determined. When the OD_450nm_ value < $\bar{x}$+2SD, it was considered as a negative serum sample. When $\bar{x}$+2SD ≤ OD_450nm_ value < $\bar{x}$+3SD, it was considered as a suspicious sample [23]. SPSS 19.0 was used to analyze whether the OD_450nm_ values of *T*. *gondii*-negative serum samples were consistent with a normal distribution.

### 2.11 Repeatability testing

The repeatability within and between runs of IMP1-iELISA was evaluated, as described previously with some modifications [24]. Six control serum samples (five *T*. *gondii*-positive mouse samples and one *T*. *gondii*-negative mouse sample) were selected for repeatability experiments. For intra-assay repeatability, each serum sample was tested in six replicates on the same plate at one time. For inter-assay repeatability, each serum sample was tested in three replicates on different plates of different days. The results were presented as the coefficient of variation (CV), which is the ratio of the SD to the mean OD_450nm_ value of each group of samples. A CV value criterion of 10% was used to meet the repeatability requirement of the test [25].

### 2.12 Cross-reaction testing

To determine the cross-reactivity, positive reference from the purchased ELISA kit for detecting the CMV, HSV, RV, *Cryptosporidium* spp., *Theileria* spp., *Neospora* spp. and *Plasmodium* spp.were tested using the IMP1-iELISA. Each sample was tested three times, and the mean OD_450nm_ value was calculated to determine whether the sample was positive or negative as described above. *T*. *gondii*-positive mouse serum and *T*. *gondii*-negative mouse serum were used as controls.

### 2.13 Sensitivity, accuracy and compliance testing

To evaluate the practicality of the IMP1-iELISA, a total of 150 mouse serum samples (90 positive serum samples and 60 negative serum samples) were evaluated by as described above IMP1-iELISA, Xishan commercial ELISA kit (Xishan ELISA) and indirect haemagglutination assay (IHA), respectively. Xishan ELISA and IHA were conducted according to the manufacturer’s instructions. For the IHA, serum samples were added to 96-well polystyrene plates, which were diluted fourfold serially from 1:4 to 1:1024. Each test was performed with positive, negative and blank controls, and serum samples which had positive reaction at dilutions of 1:64 or higher dilutions were considered positive for *T*. *gondii* antibodies [26, 27]. The results of iELISA and IHA were compared and the results of IHA were used as the standard for positive and negative to determine the sensitivity and specificity of the iELISA. Briefly, the sensitivity was defined as the ratio of positive tests from the iELISA to the positive tests from the reference IHA. The specificity was defined as the ratio of negative tests from the iELISA to the negative tests from the reference IHA.

## 3. Results

### 3.1 Epitopes of TgIMP1

**Fig 1:**
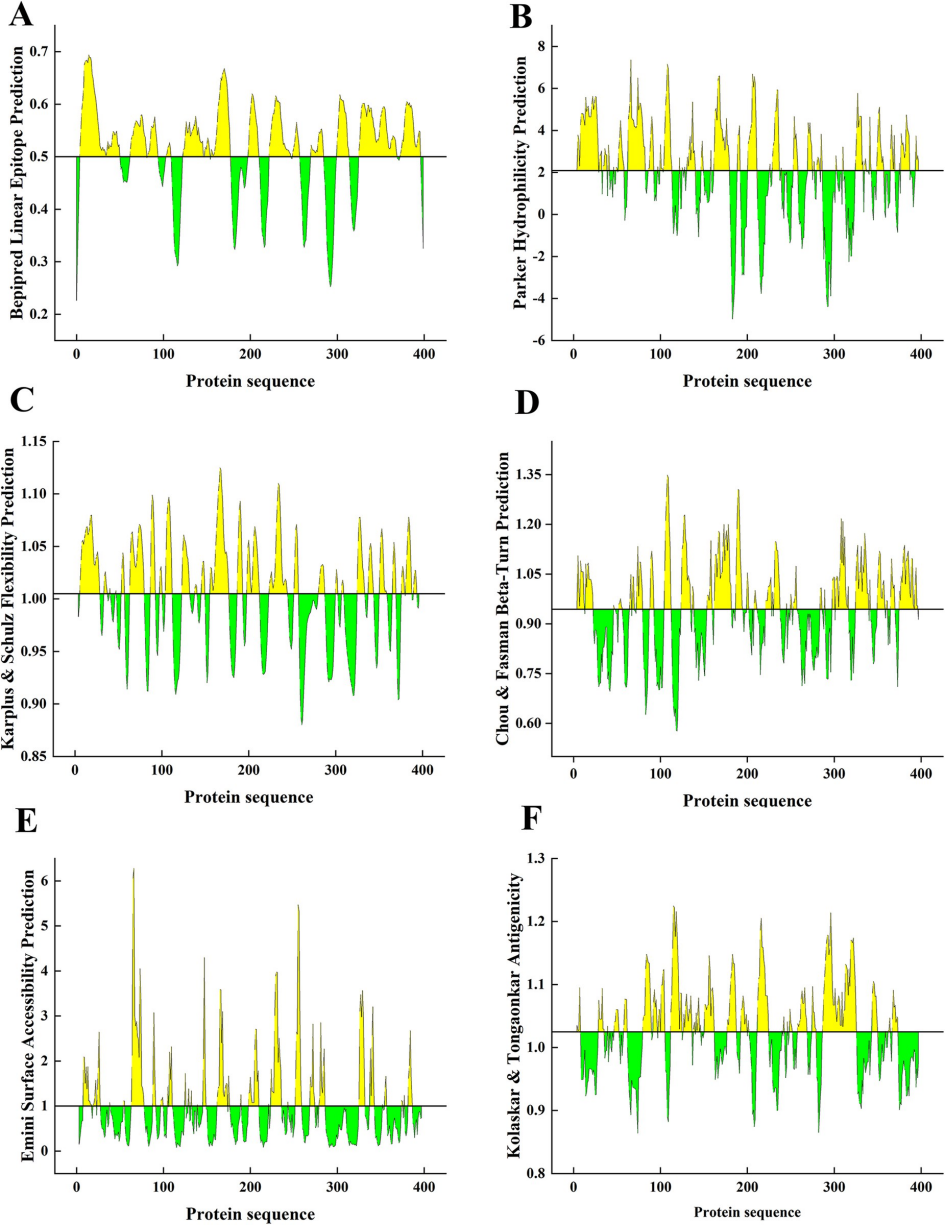
Antigenic epitope prediction of TgIMP1. The bepipred linear epitope (**A**), Parker hydrophilicity (**B**), Karplus & Schulz flexibility (**C**), Chou & Fasman beta-turn (**D**), Emini surface accessibility (**E**) and Kolaskar & Tongaonkar antigenicity (**F**) prediction of TgIMP1.

Thirteen B cell epitopes of TgIMP1 were predicted using IEDB combined with the properties of linear epitopes, β-angle, surface accessibility, skeleton flexibility, antigen index and hydrophilicity (**Fig 1**), which were located in the 5–50, 63–81, 83–94, 104–109, 124–154, 156–178, 198–210, 223–247, 250–257, 271–285, 300–314, 326–369, 374–396 amino acids, respectively (**S2 Table**).

By using ComPred online software, the threshold was adjusted to 0.5, and the binding peptides of five different allelic fragment molecules HLA-A2, HLA-A*0201, HLA-A*0202, HLA-A*0203 and HLA-A*0205 were predicted, and three dominant epitopes of CTL were screened finally. They are _44_ALTGAPAAV_52_, _181_GYLLFL_186_ and _213_VLLSFVPAL_221_ (**S3 Table**). The Th cell antigen epitopes of DRB1-0101, DRB1-0102 and DRB1-0301 alleles were predicted using the ProPred online software. The results indicated that there may be Th dominant epitopes of TgIMP1 at _217_FVPAL_221_, _298_LVGNKVASL_306_ and _373_WMKEDGIDI_381_ (**S4 Table**).

### 3.2 Construction of the pET28b-TgIMP1 prokaryotic recombinant expression plasmid

**Fig 2:**
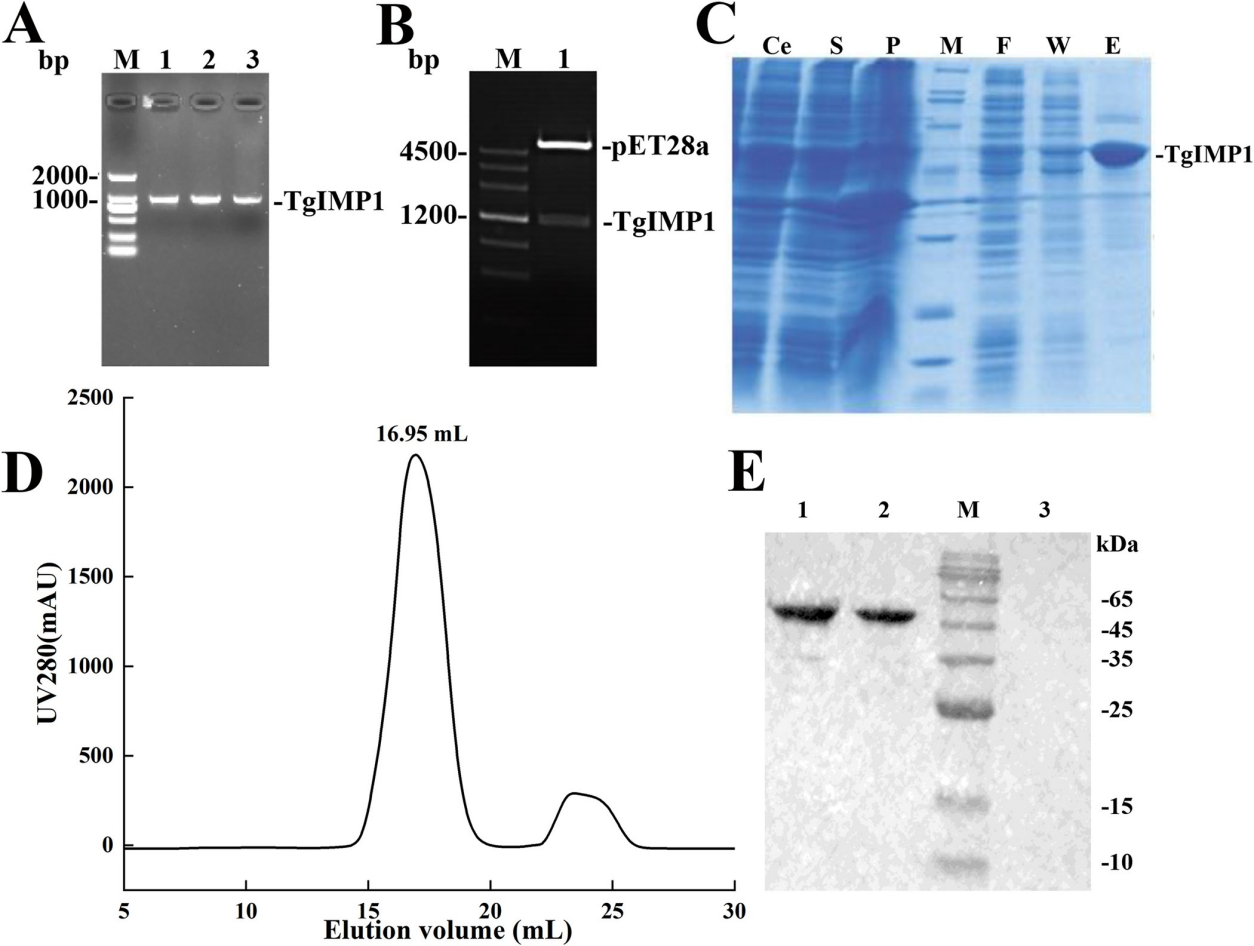
Prokaryotic expression, purification and immunogenicity identification of TgIMP1. (**A**) PCR amplification of TgIMP1. The *TgIMP1* gene has a full length of 1203 bp. (**B**) Construction and identification of TgIMP1 prokaryotic expression plasmid. (**C**) TgIMP1 purification by Ni^2+^-NTA. Ce, S, P, F, W and E were: cell lysates, soluble supernatant, insoluble precipitate, flow through liquid, washing solution and eluting solution, respectively. (**D**) The chromatograms of gel filtration of TgIMP1. The integral volume of TgIMP1 was 16.95 mL. (**E**) Identification of immunogenicity of TgIMP1. Lanes 1 and 2 were all TgIMP1 proteins obtained by Ni^2+^-NTA, and they were distinguished by their protein amounts of 0.5, 0.25 ng. Lane 3 was the negative control.

The total RNA extracted from the RH strain of *T*. *gondii* was identified by 1% agarose gel electrophoresis. Using cDNA synthesized by reverse transcription as template and TgIMP1-NdeI-PF and TgIMP1-XhoI-PR as specific primers, a specific band was obtained by PCR (**Fig 2A**). The *IMP1* gene was then attached to the pET-28b vector via T4 DNA ligase.

After sequencing identification, the plasmid was digested by NdeI and XhoI enzymes, and two bands with sizes of 5300 bp and 1200 bp were visible (**Fig 2B**), which were consistent with the sizes of pET-28b vector and IMP1, indicating that the IMP1 gene was successfully incorporated into the pET-28b vector. The positive recombinant plasmid was named pET28b-TgIMP1.

### 3.3 Expression, purification and identification of soluble TgIMP1

*E*. *coli* BL21 (DE3) strain containing pET28b-IMP1 plasmid was induced with 0.3 mM IPTG at 20°C for 12 h and purified by nickel affinity chromatography and gel filtration chromatography. The expression bands of recombinant protein were found at 45 kDa in both Ce (whole cell lysates) and S (soluble supernatant) channels by SDS-PAGE, and the intensity was the same. TgIMP1 was initially identified as a soluble expression. There were only a tiny amount of TgIMP1 protein bands in the F (flow through liquid) and W (washing solution) channels, indicating that most of the recombinant TgIMP1 fused with 6 his-tag had been bound to the Ni^2+^-NTA resin with strong affinity. The target protein was mainly in the E (elution) channel, and the impurity protein was nearly invisible to the naked eye, indicating that the purity of TgIMP1 was relatively elevated after purification by Ni^2+^-NTA column (**Fig 2C**). The chromatograms of gel filtration chromatography (**Fig 2D**) showed that the chromatographic peaks were symmetric and sharp, indicating that the TgIMP1 recombinant protein had uniform size distribution. After two steps of purification, the purity of TgIMP1 recombinant protein was more than 95% (**S1 Fig**), which means that the IMP1-iELISA has superior detection specificity. The purified TgIMP1 recombinant protein, after reacting with anti-TgIMP1 polyantibody, showed specific bands at the size of 45 kDa(**Figs 2E,and S2**), which identified the purified TgIMP1 and proved its immunogenicity.

### 3.4 Optimization of IMP1-iELISA

**Fig 3:**
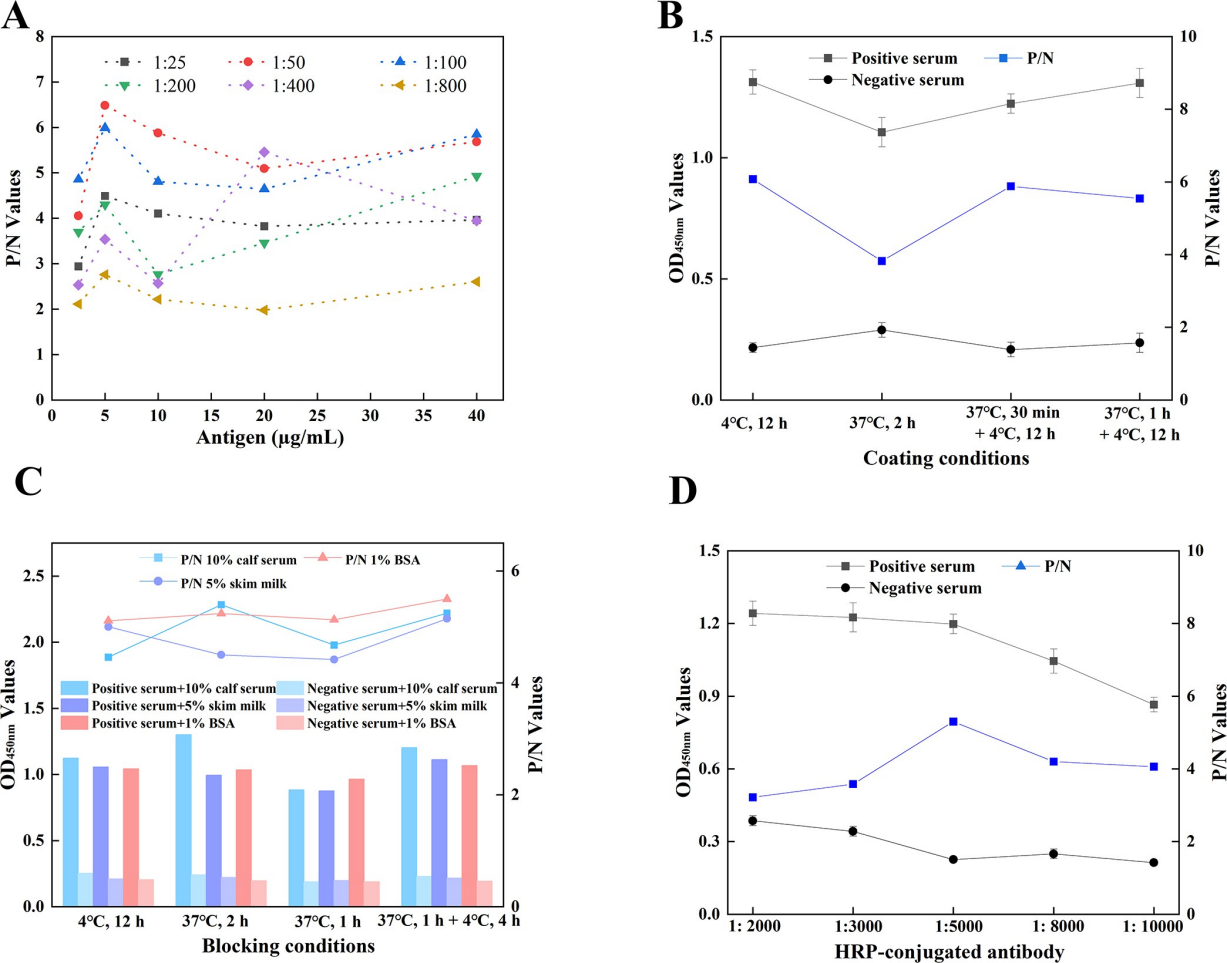
Working conditions optimization of IMP1-iELISA. The scheme with the highest P/N value was taken as the best condition. (**A**) The optimal antigen coating concentration and serum dilution. (**B**) The optimal antigen coating condition. (**C**) The optimum blocking solution and blocking condition. (**D**) The optimal working concentration of enzyme-labeled antibody.

To develop an iELISA for detecting *T*. *gondii* IgG antibodies, a checkerboard titration was performed to determine the optimal work conditions of IMP1-iELISA based on the standards that the OD_450nm_ value of positive serum was close to 1.0, the P/N value was highest, and the negative serum was low. The optimal antigen coating concentration of IMP1-iELISA was 5 μg/mL, and the optimal serum dilution was 1:50 (**Fig 3A**). Furthermore, the first antigen coating scheme (coating at 4°C for 12 h) had the highest P/N value, so it was identified as the best antigen coating condition (**Fig 3B**). After the above-mentioned conditions were determined, it was found that 10% calf serum in 1×PBST was the best blocking solution and the best blocking time for 2 h at 37°C was sufficient and time-saving in the IMP1-iELISA(**Fig 3C**). In addition, the HRP-conjugated goat anti-mouse polyclonal antibody was diluted at 1:5000 as the optimal working concentration of enzyme-labeled antibody (**Fig 3D**).

### 3.5 Criteria for judging the results of IMP1-iELISA

**Fig 4:**
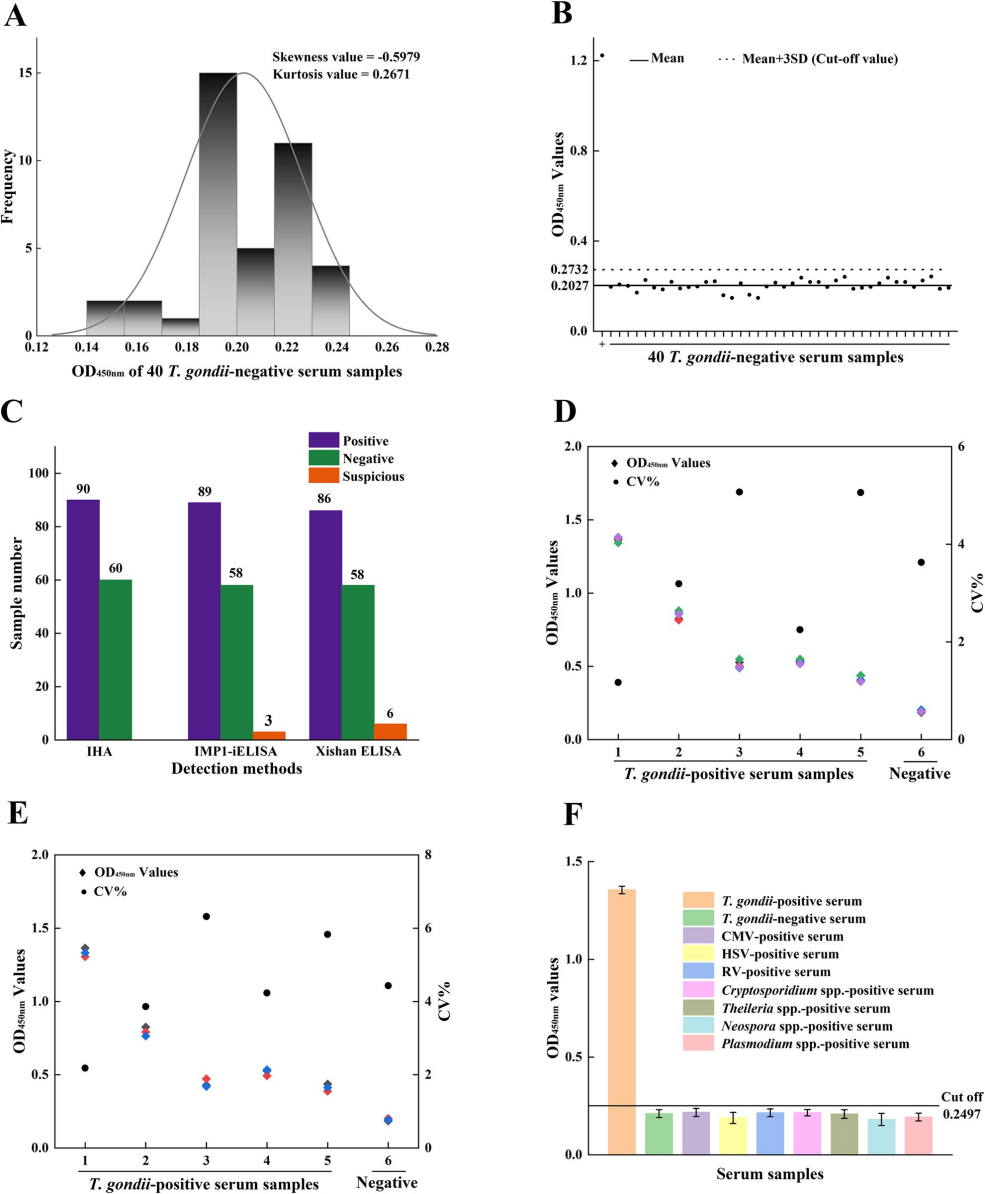
Determination of the cut-off value, repeatability and specificity of IMP1-iELISA. (**A**) Normal distribution of forty *T*. *gondii*-negative samples. The skewness and kurtosis values were calculated with SPSS 19.0. (**B**) Cut-off value of IMP1-iELISA. Forty *T*. *gondii*-negative samples were tested using the IMP1-iELISA and the mean OD_450nm_ value plus 3SD was used to calculate the cut-off value. *T*. *gondii*-positive serum as control. (**C**) IMP1-iELISA, Xishan-iELISA, and IHA were compared with 150 serum samples. (**D, E**) Six control serum samples (five *T*. *gondii*-positive samples and one *T*. *gondii*-negative sample) were tested using the IMP1-iELISA and the OD_450nm_ values of serum samples were used to calculate the CV to determine the intra-(**D**) and inter-assay (**E**) repeatability. (**F**) Cross-reaction testing of IMP1-iELISA. The data were representative of three independent experiments. Data are represented as mean ± SD.

The normal distribution analysis of forty *T*. *gondii*-negative serum samples test results showed that the skewness value was -0.5979 and kurtosis value was 0.2671, which were all less than 1, indicating that the test results obeyed normal distribution(**Fig 4A**).The mean OD value ($\bar{x}$) of the negative serum samples was 0.2027, and the standard deviation (SD) was 0.0235, $\bar{x}$+3SD = 0.2732, $\bar{x}$+2SD = 0.2497. The result determination criteria of this kit were set as follows: when the OD_450nm_ value of the sample was ≥ 0.2732, the serum was judged as a positive sample; When the OD_450nm_ value of the sample was < 0.2497, the serum was judged as a negative sample. When 0.2497 ≤ sample OD_450nm_ value < 0.2732, the serum was considered as a suspicious sample(**Fig 4B**).

### 3.6 Validation of IMP1-iELISA

To analyze the specificity and sensitivity of IMP1-iELISA, a total of 150 serum mouse samples were evaluated using IMP1-iELISA, Xishan-ELISA and IHA simultaneously. The IHA detected 90 *T*. *gondii*-positive samples, of which 89 were tested *T*. *gondii*-positive by IMP1-iELISA and 86 by Xishan ELISA. On the other hand, of the remaining 60 samples that tested *T*. *gondii*-negative by IHA, 58 of them were tested *T*. *gondii*-negative by IMP1-iELISA and 58 by Xishan ELISA. Thus, using IHA as the standard evaluation method, the sensitivity of IMP1-iELISA and Xishan ELISA to *T*. *gondii*-positive individuals was 98.9% and 95.6%, respectively, and the specificity of both IMP1-iELISA and Xishan ELISA to *T*. *gondii*-negative individuals was 96.7%. Hence, the overall coincidence rate of the IMP1-iELISA and Xishan ELISA to IHA was 98.0% and 96.0%, respectively(**Fig 4C**). These results demonstrated the excellent detection performance of IMP1-iELISA.

### 3.7 Repeatability and cross-reaction of IMP1-iELISA

In the repeatability experiment, six samples (five *T*. *gondii*-positive serum samples and one *T*. *gondii*-negative serum sample) were used to determine the intra- and inter-assay CV of the IMP1-iELISA, which was 1.17%–5.07% and 2.18%–6.32%, respectively (**Fig 4D and 4E**). All of them were less than 10.0%, indicating that the intra- and inter-assay repeatability of the IMP1-iELISA was satisfactory.

To determine the cross-reaction of IMP1-iELISA, we simultaneously tested positive serum from mouse infected with *T*. *gondii*, CMV, HSV and RV according to the requirements of the People’s Republic of China Health industry standard—Diagnosis of Toxoplasmosis (WS/T486-2015) [28]. At the same time, we conducted cross-testing with positive sera of other three apicomplexan parasites (*Cryptosporidium* spp., *Theileria* spp., *Neospora* spp. and *Plasmodium* spp.) to further verify the specificity of IMP1-iELISA. Only the *T*. *gondii*-positive serum bound to the TgIMP1 protein, with an OD_450nm_ value of 1.355, showed a positive result. The average OD_450nm_ values of positive serum samples for CMV, HSV, RV, *Cryptosporidium* spp., *Theileria* spp., *Neospora* spp. and *Plasmodium* spp. were 0.217, 0.189, 0.235, 0.226, 0.229, 0.211 and 0.193 respectively. These values were all less than 0.2497, indicating that these serum samples were *T*. *gondii*-negative and non-cross-reactive with this IMP1-iELISA (**Fig 4F**).

## 4. Discussion

*T*. *gondii* infection rates are high worldwide, especially in developing countries, and most infections are inconspicuous with no obvious clinical symptoms [29]. Therefore, laboratory tests are required for diagnosis. At present, toxoplasmosis is mainly diagnosed by pathologic, immunological, and molecular biological methods, of which ELISA has become the dominant method for clinical diagnosis and epidemiological investigation of toxoplasmosis due to its high specificity, good sensitivity, and large detection flux. Development of indirect ELISA for the detection of *T*. *gondii* IgG antibodies in human serum has been difficult due to ethical constraints and strict control of clinical samples in hospitals. Humans and mice share many genes and cell types that are important for human life, such as T cell receptors, the V, D and J gene segments of the immunoglobulin gene, toll-like receptors (TLR) and major histocompatibility complexes (MHC). The mice model has become an important vector for the study of human immunological diseases. Therefore, it is possible to first develop a method for detecting *T*. *gondii* in mice and conduct related vaccination experiments to assess the potential applications of the selected target protein in detection and vaccine protection. The rationale for ELISA is based on the specific response between antigen and antibody. Therefore, the key to establish a specific ELISA method for detecting antibodies is the selection of appropriate coat antigens [30]. In past studies, many recombinant antigens, including TgSRS2, MIC2, MIC3, MIC4, MIC5, ROP1, ROP2, ROP18, GRA3, GRA6, GRA7, GRA15, SAG1, SAG2 and MAG1 have been expressed in *E*. *coli* or yeast, and their value in the diagnosis of *T*. *gondii* has been demonstrated by ELISA [31]. TgIMP1, localized in the surface membrane of *T*. *gondii*, has been proved to be a more effective ideal antigen with strong immunogenicity, and has been used as a vaccine candidate against toxoplasmosis. However, no relevant studies have used this protein as a diagnostic antigen to establish a detection method.

In this study, the high purity TgIMP1 protein of *T*. *gondii* was obtained and then the optimal antigen coating concentration, coating condition, serum sample dilution, blocking solution, blocking condition, and working concentration of HRP-conjugated antibody were determined by checkerboard titration. Finally, a highly specific and sensitive IMP1-iELISA was established. The IMP1-iELISA does not cross-react with CMV, HSV, RV, *Cryptosporidium* spp., *Theileria* spp., *Neospora* spp. and *Plasmodium* spp., showing satisfactory specificity. The CV is less than 10% for both intra-batch and inter-batch replicates, indicating satisfactory repeatability. IMP1-iELISA has higher sensitivity and specificity compared to the Xishan ELISA kit. Compared with the traditional iELISA using a variety of antigens, the biggest advantage of IMP1-iELISA is that TgIMP1 has better physicochemical properties and stability, and a large number of TgIMP1 proteins with high purity and immunogenicity can be obtained through heteroexpression in *E*. *coli* and two-step affinity chromatography, so the detection cost will be significantly reduced. We did not compare the performance of IMP1-iELISA and SAG1-iELISA. However, recombinant TgSAG1 can be problematic to express in *E*. *coli* and also may not mimic the native protein conformation [32]. This difficulty is due to the fact that the six disulfide bonds required for proper conformation strongly affect immunological recognition [33, 34]. Therefore, the stability of the production process and the preservation of IMP1-iELISA may be better. The development of IMP1-iELISA provides a new method and technical support for the investigation, prevention and control of toxoplasmosis, and is expected to reduce the impact of toxoplasmosis on public health security. However, there are still several important issues that need to be addressed. For instance, whether the products of TgIMP1 expressed by fusion with other *T*. *gondii* proteins be used as coat antigens to further improve the detection sensitivity and specificity? How can IMP1-iELISA be applied to microfluidic chips to facilitate automated detection and improve the efficiency of large-scale sample screening? The elucidation of these questions will help us to develop a specific ELISA method for the diagnosis of toxoplasmosis.

In summary, this study has established an indirect ELISA method for the detection of *T*. *gondii* infection in mice using the recombinant TgIMP1 protein. The satisfactory specificity, sensitivity, and repeatability of this method demonstrate the potential of TgIMP1 protein in the diagnosis of toxoplasmosis and the evaluation of vaccine effectiveness.

## Supporting information

S1 Fig Purity identification of TgIMP1. The SDS-PAGE was performed on the 12.5% polyacrylamide gel.
(TIF)

S2 Fig Identification of immunogenicity of TgIMP1. The first antibody used was rabbit anti-TgIMP1 antibody, and the second antibody was horseradish peroxidase (HRP)-conjugated goat anti-rabbit polyclonal antibody. Lanes 1–8 were all TgIMP1 proteins, and they were distinguished by their protein amounts of 2, 1.75, 1.5, 1.25, 1, 0.75, 0.5, 0.25 ng. Lane 9 was the negative control.
(TIF)

S1 Table Sequences of primers used in the application of T. gondii RH strain IMP1 gene. (DOCX)

S2 Table Prediction result of B cell linear epitopes of TgIMP1. (DOCX)

S3 Table Prediction results of CTL cell epitopes of TgIMP1. (DOCX)

S4 Table Prediction results of Th cell epitopes of TgIMP1. (DOCX)
