# Supplementary figures and images for: Development of an indirect ELISA for detecting Toxoplasma gondii IgG antibodies based on a recombinant TgIMP1 protein
Source: PLoS Negl Trop Dis. 2024 Aug 14;18(8):e0012421. doi: 10.1371/journal.pntd.0012421 (PMC11346964; doi:10.1371/journal.pntd.0012421)

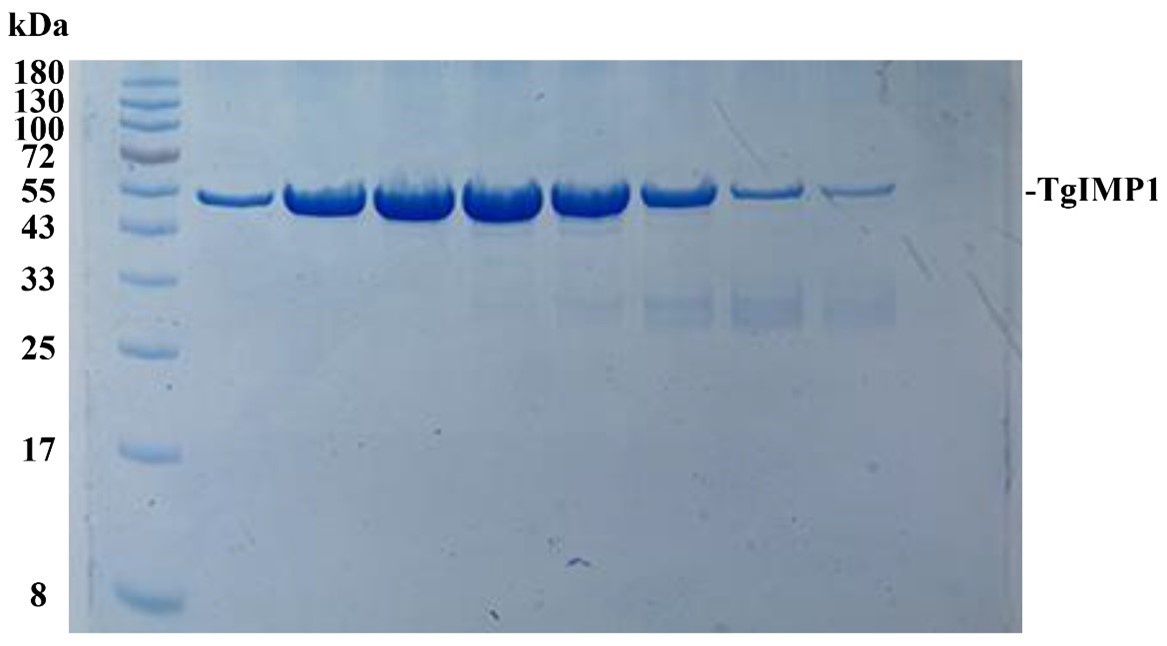

Supplement: S1 Fig — The SDS-PAGE was performed on the 12.5% polyacrylamide gel. (TIF) [file pntd.0012421.s001.tif]

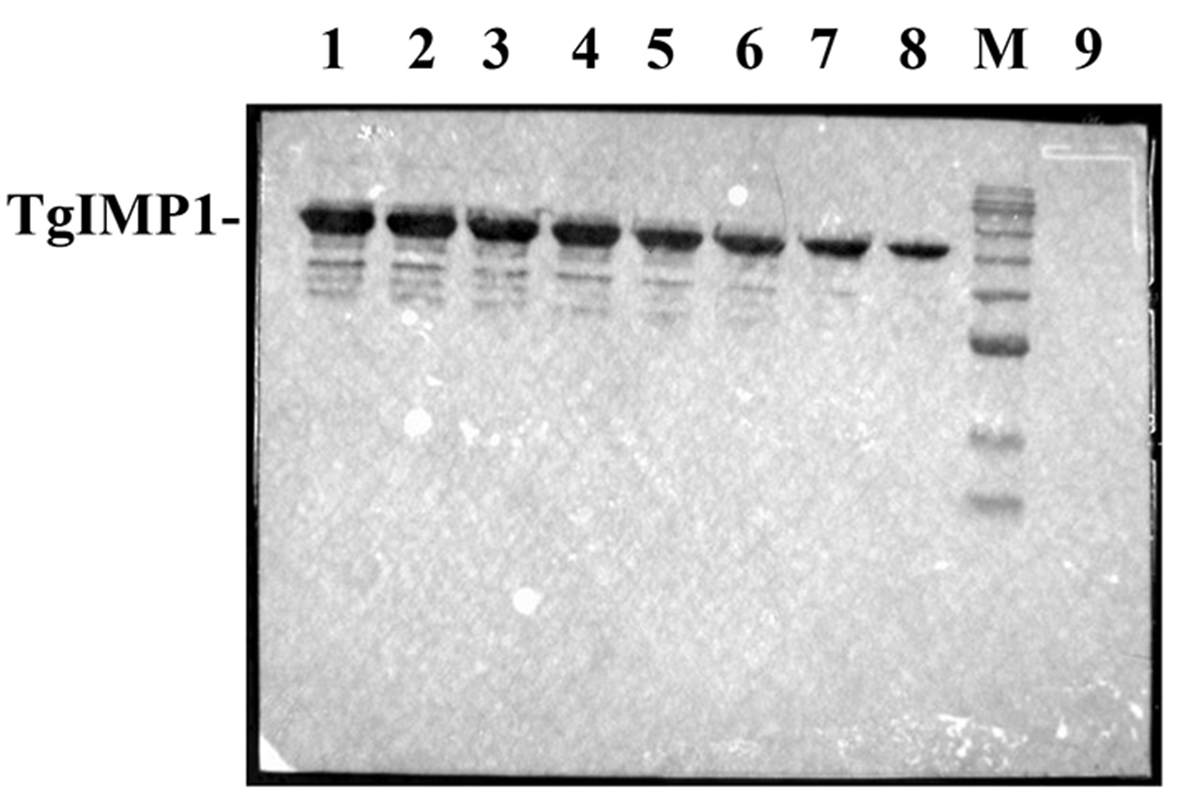

Supplement: S2 Fig — The first antibody used was rabbit anti-TgIMP1 antibody, and the second antibody was horseradish peroxidase (HRP)-conjugated goat anti-rabbit polyclonal antibody. Lanes 1–8 were all TgIMP1 proteins, and they were distinguished by their protein amounts of 2, 1.75, 1.5, 1.25, 1, 0.75, 0.5, 0.25 ng. Lane 9 was the negative control. (TIF) [file pntd.0012421.s002.tif]
