# Supplementary material for: Development of an indirect ELISA for detecting Toxoplasma gondii IgG antibodies based on a recombinant TgIMP1 protein
Source: PLoS Negl Trop Dis. 2024 Aug 14;18(8):e0012421. doi: 10.1371/journal.pntd.0012421 (PMC11346964; doi:10.1371/journal.pntd.0012421)
Supplement: S1 Table — (DOCX) [file pntd.0012421.s003.docx]

S1 Table

## Supplementary Tables

S1 Table. Sequences of primers used in the application of *T. gondii* RH strain IMP1 gene

| Primers | Sequence |
| --- | --- |
| TgIMP1-NdeI-PF | 5'- GGAATTCCATATGGCTGACGAGGCTGAGCGAACAG -3' |
| TgIMP1-XhoI-PR | 5'- TTTCTCGAGGTCCACCATTCGGCCATCAAG -3' |

Note: The underlined nucleotides are restriction sites and protective bases.
