# Supplementary material for: Development of an indirect ELISA for detecting Toxoplasma gondii IgG antibodies based on a recombinant TgIMP1 protein
Source: PLoS Negl Trop Dis. 2024 Aug 14;18(8):e0012421. doi: 10.1371/journal.pntd.0012421 (PMC11346964; doi:10.1371/journal.pntd.0012421)
Supplement: S2 Table — (DOCX) [file pntd.0012421.s004.docx]

S2 Table. Prediction result of B cell linear epitopes of TgIMP1

| No. | Start | End | Peptide | Length |
| --- | --- | --- | --- | --- |
| 1 | 5 | 50 | CTKNRVDTTKTAGTSKATADEAE  RTVAVEEGLKAAKEIEALTGAPA | 46 |
| 2 | 63 | 81 | DEEDRKQLNEERTNSVDEA | 19 |
| 3 | 83 | 94 | AAVVQQSPEPAA | 12 |
| 4 | 104 | 109 | VKSDNG | 6 |
| 5 | 124 | 154 | SDLPDLPDHVENVSAEDLELLRQARKQVMAV | 31 |
| 6 | 156 | 178 | GPVVTDITKSDQQTSQINKPSAS | 23 |
| 7 | 198 | 210 | SKQQLSAEEEENA | 13 |
| 8 | 223 | 247 | KNVPRMKYEKKGGKTELLTDIEAKW | 25 |
| 9 | 250 | 257 | WKVNEKQR | 8 |
| 10 | 271 | 285 | EYEAKVTVREWTEEM | 15 |
| 11 | 300 | 314 | GNKVASLPRGHPVDL | 15 |
| 12 | 326 | 369 | DKNKEFKDGFNLSEKKFQDLAV  AAGGADQRFAPRGIATALGQDD | 44 |
| 13 | 374 | 396 | MKEDGIDISKNERGLTLDGRMVD | 23 |
