# Supplementary material for: Development of an indirect ELISA for detecting Toxoplasma gondii IgG antibodies based on a recombinant TgIMP1 protein
Source: PLoS Negl Trop Dis. 2024 Aug 14;18(8):e0012421. doi: 10.1371/journal.pntd.0012421 (PMC11346964; doi:10.1371/journal.pntd.0012421)
Supplement: S3 Table — (DOCX) [file pntd.0012421.s005.docx]

S3 Table. Prediction results of CTL cell epitopes of TgIMP1

| Allelic fragment | No. | Start | Sequence |
| --- | --- | --- | --- |
| HLA-A2 | 1 | 44 | ALTGAPAAV |
|  | 2 | 181 | GYLLFLPDK |
|  | 3 | 213 | VLLSFVPAL |
| HLA-A*0201 | 1 | 44 | ALTGAPAAV |
|  | 2 | 213 | VLLSFVPAL |
| HLA-A*0202 | 1 | 44 | ALTGAPAAV |
|  | 2 | 143 | LLRQARKQV |
|  | 3 | 178 | SAYGYLLFL |
| HLA-A*0203 | 1 | 178 | SAYGYLLFL |
| HLA-A*0205 | 1 | 43 | EALTGAPAAVT |
|  | 2 | 44 | ALTGAPAAVT |
|  | 3 | 45 | LTGAPAAVT |
|  | 4 | 87 | QQSPEPAAV |
|  | 5 | 125 | DLPDLPDHV |
|  | 6 | 153 | AVGGPVVTD |
|  | 7 | 213 | VLLSFVP |
|  | 8 | 220 | ALHKNVPRM |
|  | 9 | 258 | YYAAWATVL |
|  | 10 | 281 | WTEEMPPQVFISLLHVGL |
|  | 11 | 284 | EMPPQVFISLLHVGL |
|  | 12 | 290 | FISLLHVGL |
|  | 13 | 304 | ASLPRGHPV |
|  | 14 | 317 | FSHIAVVPA |
|  | 15 | 356 | FAPRGIATAL |
|  | 16 | 390 | LDGRMVDRA |
