# Supplementary material for: Development of an indirect ELISA for detecting Toxoplasma gondii IgG antibodies based on a recombinant TgIMP1 protein
Source: PLoS Negl Trop Dis. 2024 Aug 14;18(8):e0012421. doi: 10.1371/journal.pntd.0012421 (PMC11346964; doi:10.1371/journal.pntd.0012421)
Supplement: S4 Table — (DOCX) [file pntd.0012421.s006.docx]

S4 Table. Prediction results of Th cell epitopes of TgIMP1

| Allele | No. | Start | Sequence |
| --- | --- | --- | --- |
| DRB1-0101 | 1 | 85 | VVQQSPEPA |
|  | 2 | 96 | IRKEEVVTV |
|  | 3 | 217 | FVPALHKNV |
|  | 4 | 258 | YYAAWATVL |
|  | 5 | 290 | FISLLHVG |
|  | 6 | 298 | LVGNKVASL |
|  | 7 | 373 | WMKEDGIDI |
| DRB1-0102 | 1 | 42 | IEALTGAPA |
|  | 2 | 85 | VVQQSPEPA |
|  | 3 | 96 | IRKEEVVTV |
|  | 4 | 151 | VMAVGGPVV |
|  | 5 | 213 | VLLSFVPAL |
|  | 6 | 290 | FISLLHVG |
|  | 7 | 298 | LVGNKVASL |
|  | 8 | 373 | WMKEDGIDI |
| DRB1-0301 | 1 | 121 | VIRSDLPDLP |
|  | 2 | 122 | IRSDLPDLP |
|  | 3 | 144 | LRQARKQVM |
|  | 4 | 158 | VVTDITKSD |
|  | 5 | 184 | LFLPDKGGS |
|  | 6 | 213 | VLLSFVPAL |
|  | 7 | 298 | LVGNKVASL |
|  | 8 | 322 | VVPADKNKE |
|  | 9 | 365 | LGQDDVVAW |
|  | 10 | 374 | MKEDGIDIS |
|  | 11 | 388 | LTLDGRMVD |
